# The effects of anterior bite plane on temporomandibular joint and mandibular morphology

**DOI:** 10.1016/j.sdentj.2023.06.002

**Published:** 2023-06-14

**Authors:** Islamy Rahma Hutami, Shella Indri Novianty, Silvia Vera Indrawati, Alif Dewa Rinaryo, Arief Rahadian, Sandy Christiono, Shaista Afroz

**Affiliations:** a Department of Orthodontics, Faculty of Dentistry, Islamic University of Sultan Agung, Semarang 50112, Indonesia; b Graduate Program of Dentistry, Faculty of Dentistry, Islamic University of Sultan Agung, Semarang 50112, Indonesia; c Department of Biochemistry, Faculty of Medicine, Islamic University of Sultan Agung, Semarang 50112, Indonesia; d Department of Pediatric Dentistry, Faculty of Dentistry, Islamic University of Sultan Agung, Semarang 50112, Indonesia; e Department of Prosthodontics/Dental Material, Dr. Ziauddin Ahmad Dental College, Aligarh Muslim University, Aligarh 202002, India

**Keywords:** Anterior bite plane, Temporomandibular joint, Mandibular angle, Point

## Abstract

**Objectives:**

An anterior bite plane (ABP) is an orthodontic appliance that prevents posterior teeth from making contact. This appliance's functional concept is to reduce muscle activity, overcome deep overbite, and temporomandibular joint (TMJ) disorders (TMD). However, ABP treatment for malocclusion frequently results in unfavorable reversible and irreversible long-term effects. This problem presents difficulties for dentists in developing an appropriate treatment modification plan in order to achieve the best results. As a result, the goal of this study is to observe the effects of different ABP types on the TMJ and mandible.

**Materials and Methods:**

Thirty-six three-month-old male Wistar strain rats were divided into three groups: control, upper flat, and upper-lower inclined ABP. The overbite and body weight were measured. TMJ was examined histologically using hematoxylin and eosin (HE). To observe the entire mandibular bone in response to ABP, mandibular planes and angulations were measured.

**Results:**

After 7 days, the upper-lower inclined ABP group has significantly lower body weight than the control group. On days 7 and 14, overbite was significantly reduced in both the upper flat and upper-lower inclined ABP groups. The superficial layer of the condyle was depleted in both ABP groups, according to HE analysis. Mandibular angle analysis revealed that the upper-lower inclined ABP group had a greater incisal and ramus angle. Furthermore, lower incisor (Li)-condyle (Co) mandibular points increased significantly more in the upper-lower inclined ABP group than in the control group.

**Conclusion:**

According to this study, various forms of ABP may have an impact on the TMJ and mandibular morphology, specifically on the length, angulation, and superficial surface of the condyle.

## 1 Introduction

Bite plane (BP) is often used to treat patients with oral parafunction and temporomandibular joint (TMJ) disorders (TMD) (Widmalm 1999). One BP modification is the anterior bite plane (ABP). Over time, the design for the ABP varies significantly according to the goals and needs of orthodontic treatment, including the mini-ABP and anterior repositioning appliance for orthopaedic purposes (Klasser and Greene 2009).

There are certain risks and contraindications for the common use of ABP, such as joint pain, increase TMD symptoms, and the eruption of posterior teeth or intrusions of anterior teeth, if used for > 2 months without appropriate supervision. Moreover, if this treatment is continued beyond the required duration, it may lead to unfavorable conditions, such as reversible or irreversible changes in the bone structure or the teeth (Widmalm 1999).

A previous study showed that modification of ABP’s band materials fixed to the maxillary incisor where the posterior teeth can still be occluded stimulating intermittent posterior condyle displacement, resulted in TMJ morphological changes induced functional malocclusion that displaced the mandible posteriorly in the occluded condition (Cholasueksa et al., 2004). Another design, twin-inclined-BP, placed on the upper and lower posterior teeth effectively induced posterior movement of the mandible and flattened posterior margin of the condyle (Hua et al., 2012, Wang et al., 2019). Unilateral anterior crossbite appliances created from metal tubes bonded on the left maxillary and mandibular incisor tubes, curved 135° labially to form an inclined incisal plate, presenting a possibility of extra loading during incising and chewing on the TMJ of the mice (Zhang et al., 2013). This appliance had a deleterious effect on the TMJ and led to the condyle's noticeable cartilage degradation (Zhang et al., 2013, Liu et al., 2014, Wang et al., 2014).

Using a flat ABP in patients with arthralgia, Torii and Chiwata (2010) were able to eliminate complaints of pain in the TMJ. However, the effect of using this tool on malocclusion is unstable (Torii and Chiwata 2010). Therefore, if the patient stops using it, the pain in the TMJ will reappear (Torii and Chiwata 2010). Moreover, a study evaluated muscular load significantly caused region-specific mandibular changes such as linear of condyle (Co) point to gnathion (Gn)-menton (Me) point and angulation of Co-gonion (Go) to Gn-Me (Hichijo et al., 2015). Excessive overbite, which is also referred to as deep overbite is a commonly encountered orthodontic problem. If untreated, it may lead to unfavourable consequences like anterior migration of maxillary anterior teeth, wear of mandibular incisors, periodontal problems, aberrations in vertical dimension, TMD and increased muscle activity (Wasinwasukul et al., 2022).

In orthodontic treatment, the excessive mechanical load on the occlusal teeth can be transmitted to the TMJ causing structural and morphological changes. Continuous changes in the structure and morphology of the TMJ can cause disorders (Dworkin et al., 1990, Koh and Robinson, 2004). Different types of BP are likely to affect the morphology of condylar surface and mechanical loading might cause morphological changes in the mandible.

The relationship between BP and TMJ remains controversial. Numerous studies have been conducted on the association between orthodontic treatment and TMJ morphological changes, but the outcome remains elusive (Huqh et al., 2020, Lin et al., 2021). Hence, more investigations are required to determine whether ABP and TMJ morphological alterations have a direct cause-and-effect relationship. Therefore, this study aimed to investigate the effects of upper flat and upper–lower inclined ABP on the TMJ as well as the change in mandibular angles and points.

## 2 Materials and methods

### 2.1 Experimental animals

Thirty-six Wistar rats (male, aged 3-months) were used for performing the experiments. Control, flat upper ABP, and upper–lower inclined ABP groups were randomly assigned to animals. Time periods of 7- and 14-day were assigned to each group. Each grouping received six animals randomly. Samples were taken from rats sacrificed 7 and 14 days later. Animal growth was tracked by body weight. All analyses were performed by a trained dentist with double-blinded investigations.

### 2.2 Design and fixation of BP

**Fig. 1:**
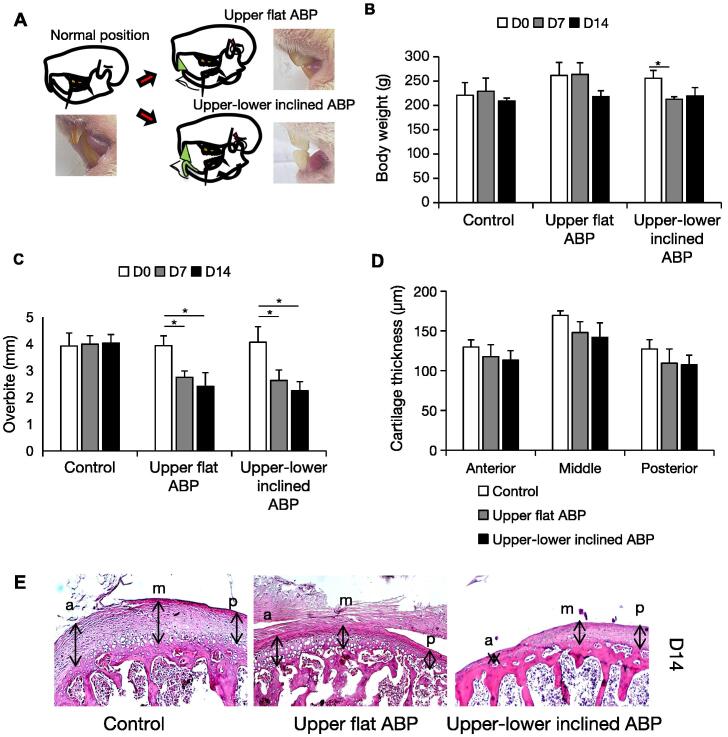
**The effects of ABP on the body weight, overbite, and condylar surface.** (a) Illustration of the mouse teeth relationship and mandible after ABP seating. The ABP was attached to the palatal surface of the upper incisal teeth and/or covered the incisal surface of the lower ones. Body weight (b) and overbite (c) of the control and experimental mice after ABP attachment (n = 6 per group). (d) Comparison of the cartilage thickness of the superficial condyle. (e) Representative sections of HE staining. Changes in the anterior (a), medial (m), and posterior (p) third thickness of the superficial condylar cartilage (n = 6 per group). Arrows point the cartilage thickness areas. Data are represented as means ± SD. Scale bar: 100 μm. *p < 0.05.

This study used two ABP variants. The first ABP was solely affixed to the upper maxillary incisor with a flat surface for the contact of mandibular incisors. This BP was fabricated from the packable composite micro-hybrid kit (3 M Z250, St. Paul, USA) and affixed to the palatal region of the first upper incisor’s incisal edge. The second ABP was a variation of the unilateral anterior crossbite appliance affixed to the bilateral upper maxillary incisors and the contact surface angled at 120° for the contact of the mandibular incisors (Zhang et al., 2013, Liu et al., 2014, Wang et al., 2014) and covering the lower first mandibular incisor’s incisal surface (Huang et al., 2011, Wu et al., 2013) for 1 mm in height (Fig. 1A).

The animals in each subgroup wore the appliance for 7 and 14 days. Effect of wearing appliance on the change in overbite was measured in the animal. All animals were terminated by cervical dislocation, after exposure to CO_2_. The right mandible was extracted and analyzed for morphometric purposes. The left mandible was preserved for histopathological examination. All experimental protocols were approved by the ethics board of Sultan Agung Islamic University (Number:216/B.1-KEPK/SA-FKG/VIII/2020) and were conducted in accordance with the Helsinki Declaration of 1975, as revised in 2013.

### 2.3 Histological analysis

TMJs were fixed in 4% paraformaldehyde and decalcified with 10% ethylenediamine-tetraacetic-acid in phosphate buffered saline for 20 days and dehydrated. Using an AEM-450 microtome (Amos Scientific, Melbourne, Australia), 5-μm-thick sagittal paraffin slices were cut from the paraffin-embedded TMJ blocks. The condylar morphology was examined using hematoxylin and eosin (HE)-stained serial paraffin slices. The thickness of the condylar cartilage was determined by separating the condylar surface into three sections: anterior, middle, and posterior (Jiao et al., 2009). The cartilage layer thickness in each place was assessed by averaging three short lines over the hypertrophic layer (Wang et al., 2014). The examination was performed under a microscope (Olympus CX23, Tokyo, Japan).

### 2.4 Mandibular morphology measurements

**Fig. 2:**
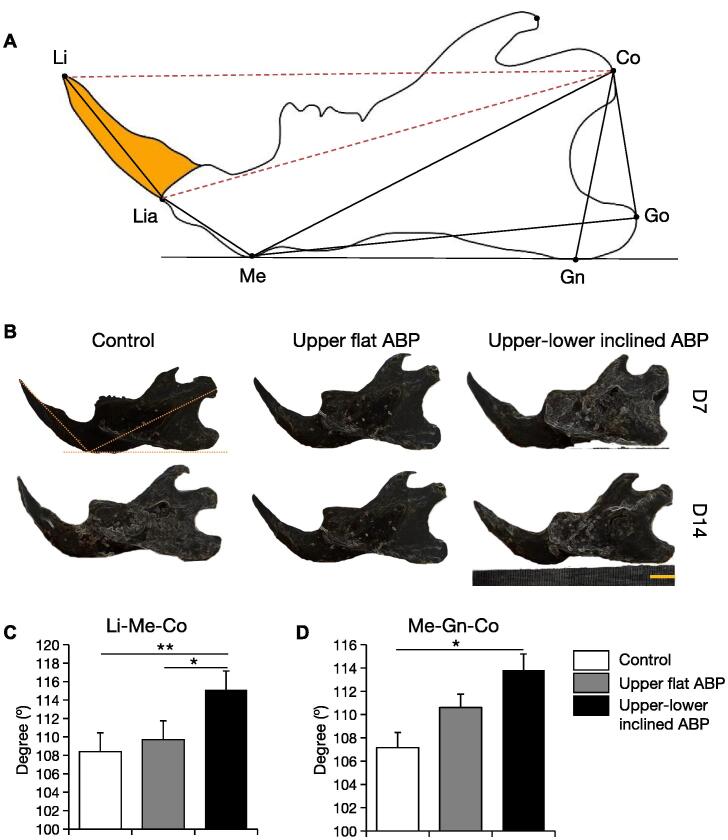
**ABP-induced angular mandibular morphological changes.** (a) Landmark and measurement items for mandibular morphology. (b) Lateral views of the mandibular morphology. (c) Incisal and (d) ramus angle measurement after 14 days of ABP treatments. Scale bar: 5 mm; n = 6 per group; **p < 0.01, *p < 0.05.

The mandibular morphology was determined with real-time images (Canon PowerShot SX530-HS, Tokyo, Japan). An orthodontist and dentists with clinical practice experience examining mandibular morphology assessed the image measurements. The images were evaluated by Image J software analysis to measure the linear and angular dimensions of the mandible. Linear and angular dimensions were assessed using six landmarks: menton (Me: most inferior point of mandibular symphysis), gnathion (Gn: most inferior contour of the mandibular angular process), gonion (Go: most outward and everted point on the angle formed by the intersection of ramus and body of mandible), condylion (Co: most superior and posterior point of mandibular condyle), lower-incisor point (Li: most upper point of the lower incisor), and lower-incisor inferior point (Lia: most inferior point of the lower incisor crown) (Hichijo et al., 2015, Ahn et al., 2019). Four linear measurements were assessed to evaluate the change in the length of the mandible: Me—Co (mandibular length), Me—Go (length of the base of the mandible), Li—Co and Lia—Co (Oksayan et al., 2014). Two linear measurements were assessed to evaluate the change in the height of the posterior mandible: Gn—Co (height of ramus) and Go—Co (Guerreiro et al., 2013). Two angular measurements used were: angle of lower incisal to the condyle (Li—Me—Co) and ramus angle (Co—Gn—Me) to determine whether this ABP design influences the growth of the mandible (Fig. 2A).

### 2.5 Statistical analysis

The results were presented as the mean ± standard deviation (SD). Analysis of variance for multiple group comparison followed by post-hoc Tukey’s honestly significant difference test between groups were performed (IBM SPSS Statistics 25.0, IBM Corp. for the software). P-values (p) < 0.01 and < 0.05 were considered statistically significant.

## 3 Results

### 3.1 ABP affects the body weight, overbite, and condylar head surface.

Animal's weight and overbite weight and overbite were measured. The upper–lower inclined ABP group lost a lot of weight after 7 days, which was maintained for the next 7 days (Fig. 1B). The upper flat and upper–lower inclined ABP groups reduced overbite 7 and 14 days after ABP insertion (Fig. 1C), with most reductions in the first week. After 14 days of ABP treatment, TMJ surface evaluation showed that the experimental groups had reduced cartilage surface than the control group in all three regions (Fig. 1D and E). Upper flat and upper–lower inclined ABPs reduce condylar surface. Together, these results suggest that dental alterations occur prior to bone changes.

### 3.2 Upper and upper–lower ABP effects on mandibular angulation and inclination

Specific lines and angles were selected to observe the effects of ABP on the mandibular morphology (Fig. 2A). After 14 days, the upper–lower inclined ABP group had a considerably different mandibular incisor angle than the control group (Fig. 2B). The upper–lower ABP group had a higher lower incisal angle Li—Me—Co than the control and upper ABP groups (Fig. 2C). Only the upper–lower ABP group had a statistically significant increase in the ramus angle Me—Gn—Co (Fig. 2D). Upper–lower inclined ABP increases lower incisor anterior inclination and ramus angles.

### 3.3 Upper and upper–lower ABP effects on mandibular morphometry

To observe the effects of both types of ABP on the mandible’s morphology, the mandible’s height and length were measured linearly. Linear measurements showed mandible length variations: Li—Co, Lia—Co, Me—Co, and Me—Go. ABP treatment did not modify mandibular base length over time. However, the upper-lower inclined ABP resulted in increase in the length of the mandible when measured at the level of Li—Co (p < 0.05) and Lia—Co (not significant). This indicates that upper–lower inclined ABP lengthened the lower incisal to the condyle without changing its location. Gn—Co and Go—Co, the posterior mandible vertical growth indicator parameters, did not differ between experimental and control groups. Thus the attenuation of the condylar surface by ABP did not result in a remarkable change in the measured distance from Co to Me, Gn, Go, and Lia.

## 4 Discussion

The BP is a bite riser worn alongside an orthodontic appliance. Acrylic or composite thickness is added on the palatal or lingual side of the anterior teeth or the occlusal part of the posterior teeth to prevent contact with the teeth in posterior or anterior region respectively (Wasinwasukul et al., 2022). The first major finding of our study was a decrease in overbite after fixing flat upper and upper-lower inclined ABP. This result agrees with the intended use of ABP to correct severe overbite, and it was accomplished within 7 days of fixing the appliance. In the group with an upper–lower inclined ABP, the lower incisal angle (Li—Me—Co) also increased. This observation is in line with the design and intent of the upper–lower inclined ABP to rectify the crossbite as the force vector of the inclined plane caused an increase in the lower incisal angle. Additional findings included attenuation of the condylar surface in both groups as well as an increase in the angle of the ramus (Me—Gn—Co) and the length of the mandible (Li—Co) in the upper–lower inclined ABP group alone.

In our study, an increase in lower incisal angle and the distance between the incisal tip of lower-incisor and condyle point without affecting the Lia point in the upper-lower inclined ABP. In studies using bite jumping appliances for the advancement of the mandible, it was observed that wearing of the appliance resulted in an increase in the length of the mandible at the level of lower incisor and mandibular condyle, which is consistent with our findings (Taira et al., 2009, Oksayan et al., 2014). An increase in the effective length of the mandible was also noted, which was not a finding in our study. The reason may be the brevity of the appliance treatment time period in our study which was 7–14 days, contrary to the observation period of their studies (Taira et al., 2009).

After 7 days, a unilateral anterior appliance causing an anterior crossbite posture caused the hypertrophic layers of the condyle to lose all calcified chondrocytes. In contrast, chondrocyte cells in the proliferation zone almost recovered to the normal TMJ at day 14 of appliance adaptation and hypertrophic layers showed an increase compared with day 7. However, in general, compared with the normal TMJ, the surface thickness of the condylar decreased quantitatively although not significantly (Zhang et al., 2013, Wang et al., 2014). Similarly, in our study, both upper flat and upper–lower inclined types of ABP led to an attenuation of the condylar surface (Fig. 1).

Functional appliances are utilized during the growth spurt to get the maximum benefit for growth modification. In our study, 3-month-old animals were used, which were in between the age groups of periadolescence and young adults and had some residual growth potential (Sengupta 2013). Many studies utilizing the BP therapy started the treatment in very young rats and continued till young adult stage (Hua et al., 2012, Hichijo et al., 2015). There are reports where ABP was used to address deep bite class II malocclusion in 15-year-old postpubertal and 19-year-old adult orthodontic patients. However, effect of the treatment on TMJ was not mentioned. In fact, the observation of the panoramic radiograph revealed that the condyle heads were asymmetrical (Dixit et al., 2016, Narmada et al., 2023). As long as orthodontic treatment is successful and produces adequate occlusion, the effect of treatment on patient's TMJ is not anticipated. Thus, it is imperative that specific observation of the TMJ is done during BP treatment.

**Fig. 3:**
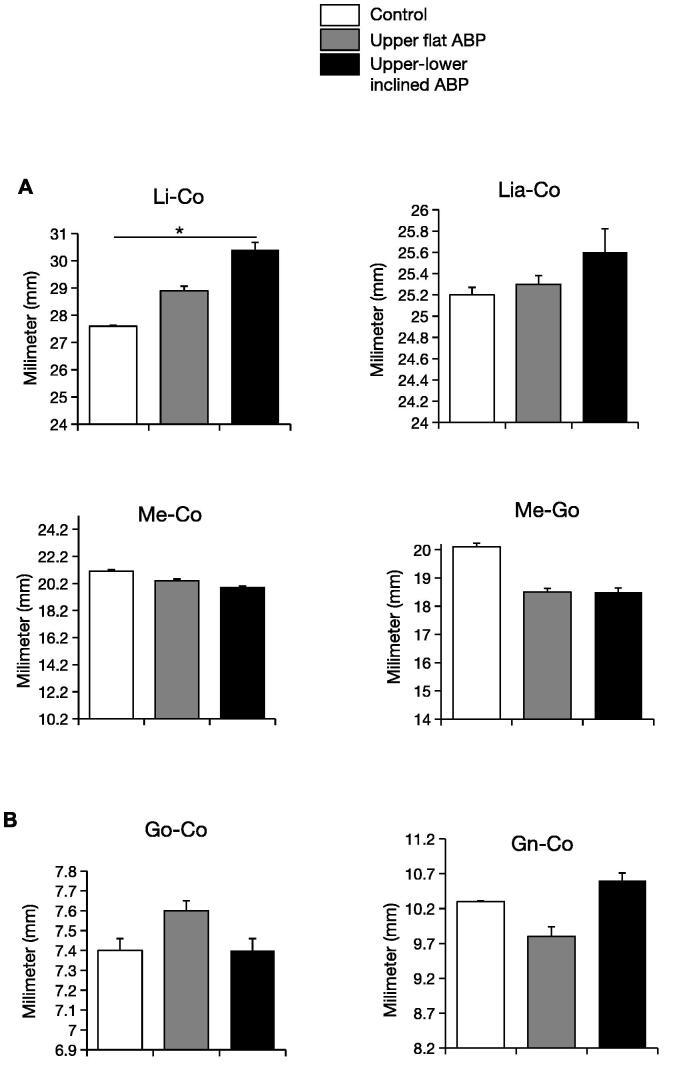
**ABP-induced linear mandibular morphological changes.** (a) Horizontal Li–Co, Lia–Co, Me–Co, and Me–Go measurements (b) vertical Go–Co and Gn–Co measurements after 14 days of ABP treatments. n = 6 per group; *p < 0.05.

As patients undergoing active orthodontic treatment with BP modification often complain of disturbances in the TMJ, it is crucial to evaluate the treatment outcome periodically to check the progress and achieve orthopedic stability of the masticatory system (Srivastava et al., 2013). Preventing adverse effects that may prolong the treatment should be considered to avoid extended orthodontic treatment (Shankland, 2004). Here we assessed the mandibular morphological effect of the ABP in normal masticatory function after 2 weeks of adaptation. The angulation of Li-Me-Co and Me-Gn-Co in the upper-lower group showed a significant increase compared to the upper and control groups (Fig. 2). In contrast, linear measurement guided by the condyle point did not demonstrate a significant difference, except Li-Co in the upper–lower inclined group (Fig. 3). These results imply that for correcting deep bite and crossbite in adolescent animals, brief intervention should be considered to avoid long-term deleterious effects.

BP therapy using either ABP or inclined plane devices affects the position of the condyle during the masticatory cycle, which leads to bone remodelling and condylar cartilage alterations (Hua et al, 2012). This explains that the forward movement of mandible in the ABP group in the present study had influenced the adaptive modeling of TMJ surface, the angulation angle of the mandible and teeth, and the linear distance of the mandibular incisal tip to the condyle. In our study, the ABP designs were chosen to elucidate on the direct effect of BP on TMJ. There are numerous ABP varieties that were utilized alone or in conjunction with orthodontic appliances (Albagieh et al., 2023). As both upper-flat and upper-lower inclined ABP in adult treatment did not promote mandibular growth but had a direct effect on the TMJ, these two models are frequently used either alone or in conjunction with orthodontic treatment (Sari et al., 2001, Showkatbakhsh et al., 2011). To overcome the limitation of our study, in future, it is necessary to analyze intracellular biomarkers of bone remodeling and long-term use of BP to determine the extent of alterations in the TMJ. Additional investigations to elucidate the detailed mechanism of mandibular movement to the maxilla during BP application is necessary to augment our understanding of TMD pathogenesis and diagnosis. Moreover, the effect of BP therapy needs to be evaluated in young animals from the prepubertal growth period until the late adolescent period to clearly understand the effect on each age group.

## 5 Conclusion

This study provides an analysis of BP modifies the TMJ and mandibular morphology. Instead, the different alterations of ABP lead to thinning of the condylar surface due to direct stress transmission to the rat’s TMJ. In addition, the morphology of the mandible only affects the position of the lower incisor point to the condyle.
